# Effects of Moisture Diffusion on a System-in-Package Module by Moisture–Thermal–Mechanical-Coupled Finite Element Modeling

**DOI:** 10.3390/mi13101704

**Published:** 2022-10-10

**Authors:** Zhiwen Chen, Zheng Feng, Meng Ruan, Guoliang Xu, Li Liu

**Affiliations:** 1 The Institute of Technological Science, Wuhan University, Wuhan 430072, China; 2 Tsinghua Innovation Center in Zhuhai, Zhuhai 519080, China; 3 School of Materials Science and Engineering, Wuhan University of Technology, Wuhan 430070, China

**Keywords:** epoxy molding compound, hygroscopic test, moisture distribution, stress distribution, finite element modeling

## Abstract

Epoxy molding compounds (EMCs) are commonly used in electronic products for chip encapsulation, but the moisture absorption of EMC can induce significant reliability challenges. In this study, the effects of hygrothermal conditions and structure parameters on moisture diffusion and the consequent influences (such as moisture content on die surfaces and stress distribution) on a system-in-package module have been systematically investigated by moisture–thermal–mechanical-coupled modeling. Hygroscopic tests were carried out on a new commercial EMC at 60 °C/60% RH and 85 °C/85% RH, followed by evaluations of diffusion coefficients by Fick’s law. It was found that the moisture diffusion coefficients and saturation concentrations at 85 °C/85% RH were higher than those at 60 °C/60% RH. From the modeling, it was found that the consequent maximum out-of-plane deformation and stress of the module at 85 °C/85% RH were both higher than those at 60 °C/60% RH. Influences of thicknesses of EMC and PCB on the moisture diffusion behavior have also been studied for design optimization. It was found that the maximum moisture concentration on die surfaces and resultant stress increased notably with thinner PCB, whereas the effects of EMC thickness were limited. This can be attributed to the comparison between the thicknesses of EMC and PCB and the shortest existing diffusion path within the module. These findings can provide helpful insights to the design optimization of electronic modules for hygrothermal conditions.

## 1. Introduction

Epoxy molding compounds (EMCs) are widely used in consumer products to protect encapsulated devices. However, a notable side effect of EMCs is the moisture absorption from the air until saturation. This can pose serious threats to the performance and reliability of a device due to potential “pop-corning” failure, swelling, electrochemical migration, and interfacial degradation [1,2,3].

In the literature, various methodologies were reported to characterize the effects of moisture absorption on the mechanical performance of bulk EMC and EMC/substrate interfaces, such as piezoresistive stress sensor chips for strain measurements [4], and micro-digital image speckle correlation (μ-DiSC) for interfacial fracture toughness [5]. Finite element modeling and molecular dynamics were also frequently used to study the influences of moisture absorption on the device reliability [6,7,8,9,10,11]. It was found that the interactions between water molecules and the chain structure of epoxy polymer during moisture diffusion can alter the mechanical and thermal performance of EMCs [7,12,13]. However, the majority of recent studies have focused on the diffusion behavior of moisture [12,13,14,15,16]. The interactions between electronic modules and moisture diffusion have seldom been studied, such as resultant stress distribution by moisture diffusion, and the effects of structure parameters of an electronic module on moisture diffusion.

In this study, a new commercial EMC was studied for hygroscopicity under different conditions: 85 °C/85% RH and 60 °C/60% RH. After hygroscopic tests, a moisture–thermal–mechanical-coupled model was built for a system-in-package module to investigate the effects of hygrothermal conditions on the moisture and stress distribution. Influences of major structure parameters of the module on the stress and moisture distribution were also studied, such as the thickness of the EMC and the printed circuit board (PCB).

## 2. Materials and Methods

**Figure 1:**
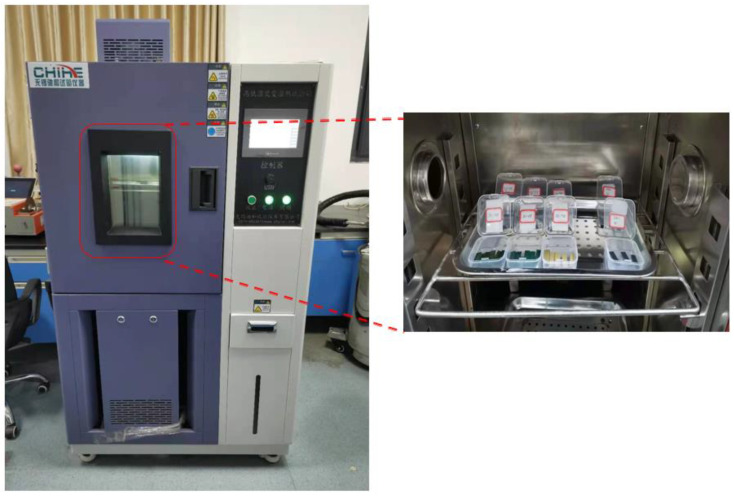
Hygroscopic tests on EMC samples.

EMC strips with of 30 mm × 8 mm × 0.8 mm (length × width × thickness) in size were prepared for hygroscopic tests. According to JEDEC J-STD-020E [17], the samples were baked in an oven at 125 °C for 24 h to minimize the effects of residual moisture and obtain the dry weights before tests. Subsequently, the samples were tested in a temperature and humidity chamber (CHTH4005, temperature range: −40 °C–150 °C, humidity range: 10–98% RH) at two different hygrothermal conditions: 85 °C/85% RH and 60 °C/60% RH for 50 h (Figure 1). Five samples were tested for each condition to obtain more reliable results. Samples were weighed with a high-precision electronic balance (accuracy 0.1 mg) before hygroscopic tests, and every 4 h after the tests started.

**Figure 2:**
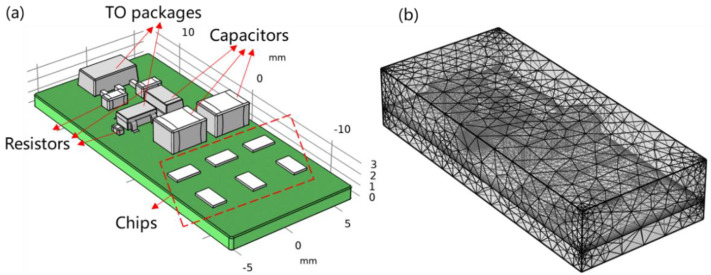
Geometry of the model: (**a**) schematic of the model with molding compound hidden; (**b**) mesh of the model (122,857 elements).

To further elucidate the effects of moisture absorption on the reliability of electronic modules, a finite element model for a system-in-package module was built, as shown in Figure 2. Diffusion coefficients and saturation concentrations of EMCs were obtained from hygroscopic tests. Other parameters were provided by the vendor or from the literature, as listed in Table 1.

Based on the model in Figure 2, the bottom center of PCB was fixed with no other mechanical constraints. The transport of diluted species module in Comsol was used to simulate the moisture diffusion behavior [22]. Moisture diffusion within both EMC and PCB was included in the model with compliance to Fick’s law. Two sets of hygrothermal loads were applied in modeling: 85 °C/85% RH and 60 °C/60% RH for 168 h (case 1 and case 2 in Table 2, respectively). To simulate the effects of moisture diffusion on an electronic module, two other physical modules (such as solid mechanics and heat transfer) and the corresponding coupling modules (such as thermal expansion and hygroscopic swelling) were utilized to investigate the coupling effects of temperature, moisture, and solid mechanics under hygrothermal conditions. The governing theories of these phenomena in modeling can be expressed as follows [22]:

(1) Theory for heat transfer:

(1) $${\rho C}_{p}\left(\frac{\partial T}{\partial t}+u_{\mathrm{trans}}\cdot \nabla T\right)+\nabla \cdot \left(q+q_{r}\right)=-\alpha T:\frac{\mathrm{dS}}{\mathrm{dt}}+Q$$

where ρ is the density, C_p_ is the specific heat capacity at constant stress, T is the absolute temperature, **u**_trans_ is the velocity vector of translational motion, **q** is the heat flux by conduction, **q**_r_ is the heat flux by radiation, α is the coefficient of thermal expansion, S is the second Piola–Kirchhoff stress tensor, and Q contains additional heat sources.

(2) Theory for the transport of diluted species by Fick’s law approximation:

(2) $$j_{\mathrm{md},i}=-\rho_{i}D_{i,\mathrm{kl}}^{F}\frac{\nabla x_{i}}{x_{i}}$$

where j_md,i_ is the diffusive flux, ρ_i_ is the density, x_i_ is the mole fraction of species i, and $D_{i,kl}^{F}$ represents a general diffusion matrix.

(3) Theory for structural mechanics:

(3) σ=C:ε

where **σ** is the stress tensor, **ε** is the strain tensor, and **C** is the constitutive tensor.

(4) Theory for thermal expansion:

(4) $$\varepsilon_{\mathrm{th}}=\alpha \left(T-T_{\mathrm{ref}}\right)$$

where ε_th_ is the strain by thermal expansion, α is the coefficient of thermal expansion, and T and T_ref_ are the current temperature and reference temperature, respectively.

(5) Theory for hygroscopic swelling:

(5) $$\varepsilon_{\mathrm{hs}}=\beta_{h}M_{m}\left(c_{\mathrm{mo}}-c_{\mathrm{mo},\mathrm{ref}}\right)$$

where β_h_ is the coefficient of hygroscopic swelling, M_m_ is the molar mass, c_mo_ is the moisture concentration, and c_mo,ref_ is the strain-free reference concentration.

Some hypotheses were also included to simplify the modeling: (1) after baking at 125 °C for 24 h, the moisture in EMC was assumed to be ideally removed; and (2) both PCB and EMC were assumed to be isotropic and elastic in thermal expansion and hygroscopic swelling.

To elucidate the different effects of thermal expansion and hygroscopic swelling on the stress distribution in the module, the same model was then studied at 85 °C/85% RH with only thermal expansion or hygroscopic swelling for comparison (case 3 and case 4 in Table 2). To further study the influences of structure parameters, models with different EMC thicknesses (2.7~3.7 mm) and PCB thicknesses (0.7~1.7 mm) were then investigated for the comparisons of maximum moisture concentration and stress at IC chips (case 5 and case 6 in Table 2).

## 3. Results

### 3.1. Hygroscopicity of EMC

**Figure 3:**
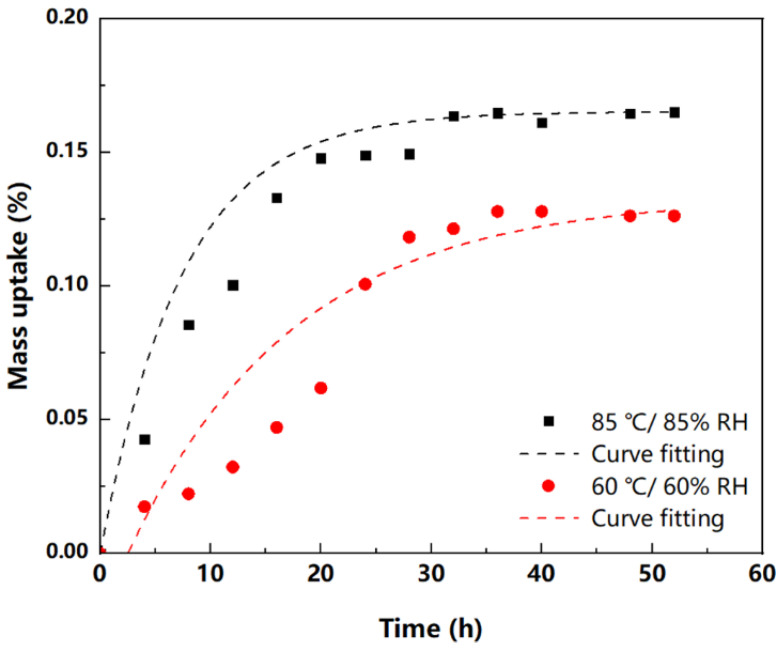
Correlation between mass uptake and time in hygroscopic test.

Figure 3 shows the average mass increases in EMC at 85 °C/85% RH and 60 °C/60% RH. For both conditions, the weight increases were faster at the beginning of tests, but generally stabilized when the tests lasted for longer than 35 h. Although different diffusion models have been proposed for moisture diffusion [23,24,25,26], Fick’s law is still the most common theory used for data analysis [12,27], which was also employed in our study.

For homogeneous materials, Fick’s law can be expressed as:

(6) $$\frac{\partial C}{\partial t}=D\left(\frac{\partial^{2}C}{\partial x^{2}}+\frac{\partial^{2}C}{\partial y^{2}}+\frac{\partial^{2}C}{\partial z^{2}}\right)$$

where D (mm^2^/s) is the diffusion coefficient and C (g/mm^3^) is the moisture concentration. For one-dimensional cases, the sample weight can be expressed as a function of time:

(7) $$\frac{M_{t}}{M_{\infty }}=1-\frac{8}{\pi^{2}}\sum_{n=0}^{\infty }\frac{1}{{\left(2n+1\right)}^{2}}\exp(\frac{-D{\left(2n+1\right)}^{2}\pi^{2}}{4l^{2}}t)$$

where M_t_ is the sample weight at time t, M_∞_ is the weight after saturation, and l is the thickness of the sample.

Based on curve fitting with Equation (7), the diffusion coefficients at 85 °C/85% RH and 60 °C/60% RH can be evaluated. The relatively higher deviation between 10 h and 20 h in Figure 3 can be attributed to the relatively lower absolute weight of absorbed moisture in the initial stage of tests. Moreover, the saturation concentration of moisture within EMCs can also be estimated by $C_{sat}=\frac{M_{\infty }}{V}$. Therefore, major moisture diffusion parameters of this new commercial EMC are listed in Table 3. In the literature, it has been reported that the diffusion coefficients for most molding compounds at 85 °C ranged from 2 × 10^−13^ m^2^/s to 6 × 10^−13^ m^2^/s [28,29,30]. The moisture diffusion coefficient of this new EMC is close to the lower bounds of reported results.

### 3.2. Influences of Hygrothermal Conditions

**Figure 4:**
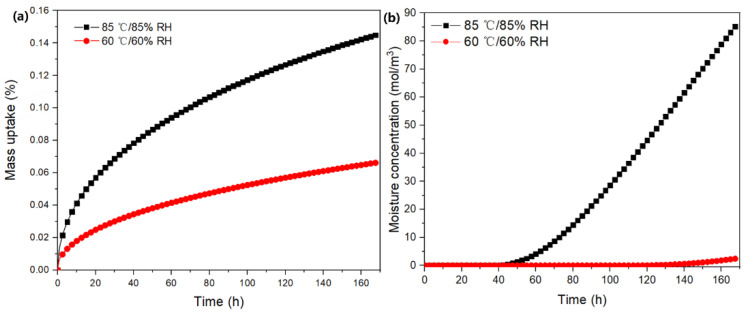
Effects of hygrothermal conditions on moisture diffusion: (**a**) mass uptake of the entire module; (**b**) evolution of the maximum moisture concentration on the chip surfaces.

To elucidate the effects of moisture absorption on electronic modules, a moisture–thermal–mechanical-coupled model was built for a system-in-package module under different conditions: 60 °C/60% RH and 85 °C/ 85% RH. Figure 4a shows that the mass uptake within the module at 85 °C/85% RH and 60 °C/60% RH generally conformed with Fick’s law, but the rate at the former was evidently higher than that at the latter. This also shows that the moisture absorption did not even reach an equilibrium state after 168 h, which can be attributed to the high volume of EMC in this module. Figure 4b illustrates the comparison of maximum moisture concentrations on die surfaces under different conditions. This shows that moisture concentrations on die surfaces remained close to 0 mol/m^3^ in the initial 40 h of diffusion. Subsequently, the increase in moisture concentration at 85 °C/85% RH was much faster than that at 60 °C/60% RH. The final content at 85 °C/85% RH was also approximately 34.6 times higher than that at 60 °C/60% RH.

**Figure 5:**
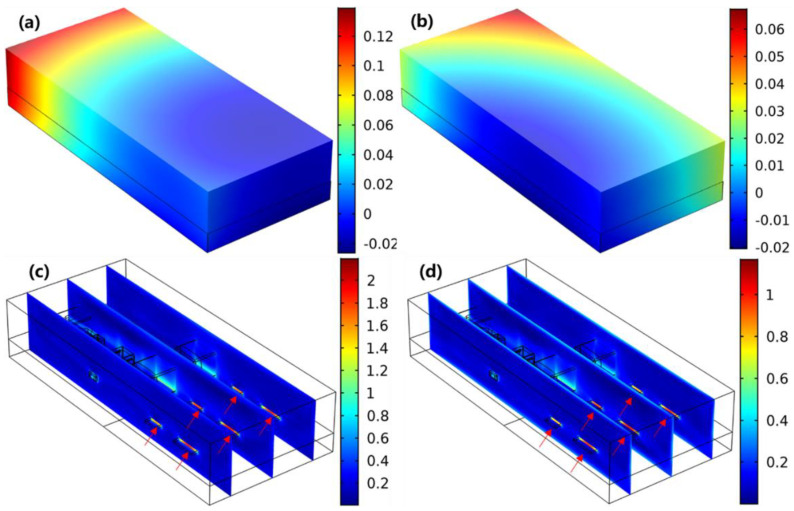
Out-of-plane deformation at (**a**) 85 °C/85% RH and (**b**) 60 °C/60% RH (unit: mm); stress distribution at (**c**) 85 °C/85% RH and (**d**) 60 °C/60% RH (unit: ×10^8^ Pa).

Figure 5a,b show the comparison of out-of-plane deformation within the module after subjected to 85 °C/85% RH and 60 °C/60% RH for 168 h. It can be found that the warpage at 85 °C/85% RH (0.14 mm) was more severe than that at 60 °C/60% RH (0.07 mm). In addition, the shape at 85 °C/85% RH was slightly different from that at 60 °C/60% RH with different locations of minimum deformation along the Z axis. Figure 5c,d illustrate the stress distribution within the module. It is shown that the stress was generally concentrated at the chips, and the maximum stress at 85 °C/85% RH (219 MPa) was much higher than that at 60 °C/60% RH (117 MPa).

**Figure 6:**
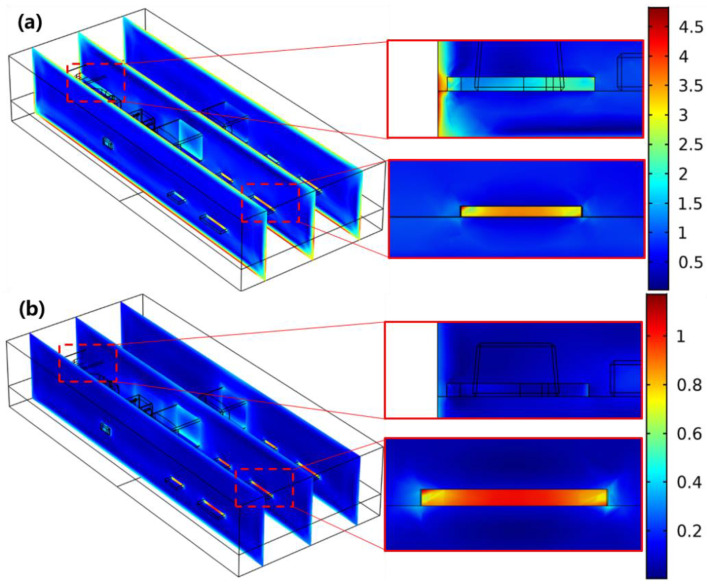
Distribution of von Mises stress by (**a**) hygroscopic swelling (unit: ×10^7^ Pa) and (**b**) CTE mismatch (unit: ×10^8^ Pa) at 85 °C/85% RH.

To compare the contributions of temperature and moisture with stress distribution within the electronic module, the models were then built with only thermal expansion or hygroscopic swelling at 85 °C/85% RH (case 3 and case 4 in Table 2). Figure 6a,b show the comparison of influences of hygroscopic swelling and mismatch of CTE on stress distribution within the module. This shows that the stress by moisture absorption was generally concentrated at both the chips and the Al leads of the TO package (as illustrated in Figure 6a). However, the maximum stress induced by the mismatch of CTE was located at the chips (Figure 6b). Both EMC and PCB were capable of absorbing moisture under hygrothermal conditions, but the moisture diffusion within Si and Al was negligible, as detailed in Table 1. This led to the mismatch of hygroscopic swelling and consequent stress concentration within both Si chips and Al leads, as depicted in Figure 6a. The stress concentration at IC chips shown in Figure 6b can be attributed to its lower thermal expansion in comparison with EMC and PCB. From Table 1, the CTE of EMC is 5.6 times higher than that of IC chips, but those of EMC, PCB, and Al are much closer to each other. Therefore, the stress by CTE mismatch was generally concentrated at the chips (Figure 6b).

### 3.3. Effects of Structure Parameters

**Figure 7:**
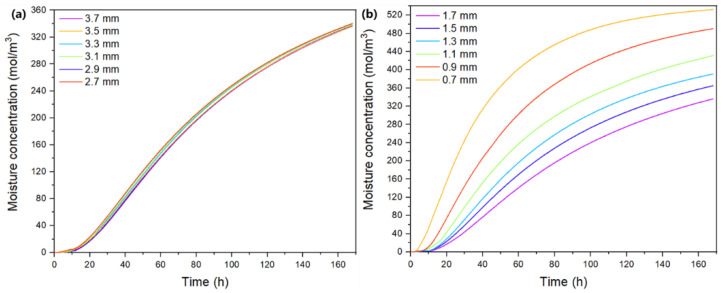
Effects of (**a**) EMC thickness and (**b**) PCB thickness on the evolution of maximum moisture concentrations on die surfaces at 85 °C/85% RH.

Figure 7 illustrates the effects of PCB thickness and EMC thickness on the maximum moisture concentration on the die surfaces in the module at 85 °C/85% RH. It shows that maximum moisture concentration on die surfaces generally increased with a thinner PCB or EMC. When the EMC thickness dropped from 3.7 mm to 2.7 mm, the maximum moisture concentration on die surfaces increased from 336.1 mol/m^3^ to 340.1 mol/m^3^. In contrast, the effects of PCB thickness are more significant. When the PCB thickness decreased by 1 mm, the highest moisture concentration on die surfaces reached about 1.6-fold higher than the initial value, from 336.1 mol/m^3^ to 532.3 mol/m^3^. This implies that the moisture concentration within this module is more sensitive to PCB thickness than EMC thickness.

**Figure 8:**
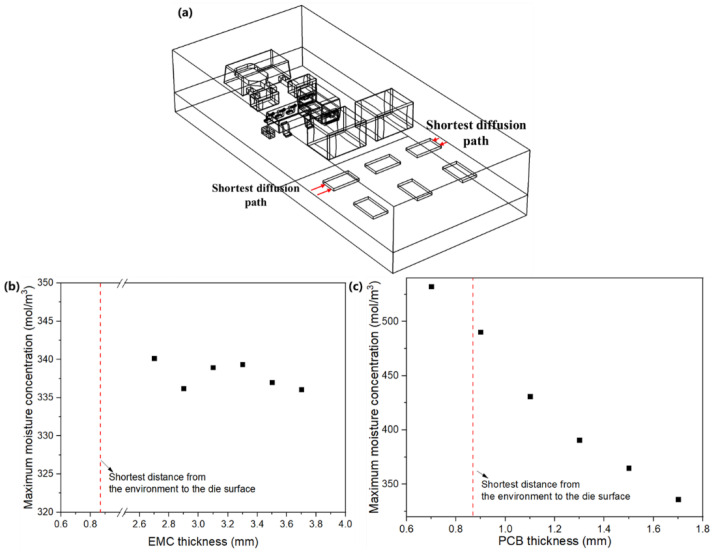
(**a**) Shortest diffusion path in the original model (case 1) and the effects of (**b**) EMC thickness and (**c**) PCB thickness on the maximum moisture concentration on die surfaces.

These changes can be attributed to the comparison of EMC and PCB thicknesses with the shortest existing diffusion path (Figure 8). In the original model (case 1), the shortest diffusion path was from the side surfaces to chips, about 0.87 mm (Figure 8a). In modeling, the investigated EMC thicknesses were far greater than the shortest existing diffusion path due to the capacitors. Therefore, reducing EMC thickness can only pose a minimal effect on the maximum moisture content on die surfaces (Figure 8b). In contrast, the investigated PCB thicknesses are close to the shortest existing diffusion distance; thus, reducing the PCB thickness can significantly enhance the moisture diffusion into the module through PCB. Hence, the maximum moisture content on die surfaces increased significantly with the thinner PCB (Figure 8c).

**Figure 9:**
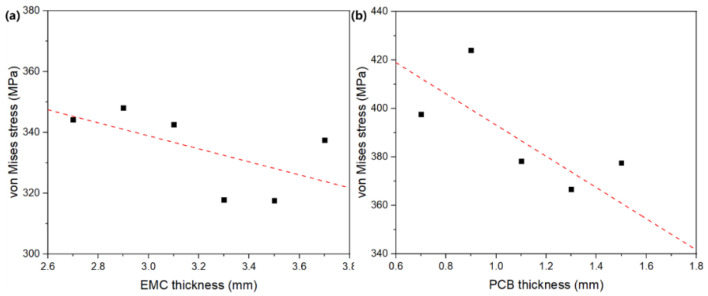
Effects of (**a**) EMC thickness and (**b**) PCB thickness on the maximum stress within IC chips under 85 °C/85% RH.

Figure 9 demonstrates the comparison of influences of EMC thickness and PCB thickness on maximum stress within silicon chips under 85 °C/85% RH. This shows that the maximum stress generally decreased linearly with a thicker EMC or PCB. Curve fitting yielded σ = 403.1-21.4d for EMC thickness and σ = 457.6-64.5d for PCB thickness, where *σ* is the maximum stress in chips (MPa) and *d* is the thickness (mm). The negative correlation between maximum stress and thicknesses of EMC and PCB can be attributed to smaller amounts of absorbed moisture and less consequent swelling mismatch if the thickness increases. The maximum stress is more sensitive to PCB thickness (Figure 9b) because it is at a comparable level to the shortest existing diffusion path.

Consequently, in module design, optimizing structural parameters (such as EMC thickness and PCB thickness) can be used to improve the stress distribution and moisture concentration on die surfaces when subjected to hygrothermal conditions. It is also essential to take the influences of the shortest existing diffusion path into account.

## 4. Conclusions

In this study, the moisture absorption behavior of a new commercial EMC was evaluated by hygroscopic tests. The effects of hygrothermal conditions and structure parameters on the stress were studied, as was moisture distribution within a system-in-package module. Based on the presented results and discussions, the following conclusions can be drawn:

1. The moisture diffusion coefficient and saturation content of the new EMC at 85 °C/85% RH was higher than the values at 60 °C/60% RH;
2. For the studied module, both the maximum out-of-plane deformation and maximum stress at 85 °C/85% RH were higher than the values at 60 °C/60% RH;
3. When subjected to hygrothermal condition, the stress caused by hygroscopic swelling generally concentrated at the chips and Al leads, but the maximum stress by CTE mismatch was located at the chips;
4. Structure parameters can pose significant effect on the distribution of stress and moisture within electronic modules under hygrothermal conditions, which is particularly true when the structure parameters are close to the shortest existing diffusion path.

In future, we will further investigate the combined effects of hygrothermal conditions, moisture diffusivity, shortest diffusion path, and structure parameters on moisture diffusion behaviors. Therefore, more generalized guidelines can be derived for design optimization.

## Figures and Tables

**Table 1:** Parameters for modeling on hygroscopic tests.

Parameters	EMC	PCB	Si	Al
Young’s modulus (MPa)	16,520 [18]	18,200 [18]	131,000 [19]	70,000 [19]
Poisson’s ratio	0.25 [18]	0.25 [18]	0.28 [19]	0.3 [19]
Coefficient of thermal expansion (CTE) (10^−6^ K^−1^)	14.8 [18]	15 [18]	2.63 [19]	21 [19]
Coefficient of hygroscopic expansion (CHE) (m^3^/kg)	4 × 10^−4^ [20]	3.5 × 10^−4^ [21]	0	0
Specific heat (J/(kg·K))	236 [18]	920 [18]	700 [19]	900 [19]
Thermal Conductivity (W/(m·K))	0.6 [18]	0.2 [18]	148.27 [19]	237 [19]
Moisture diffusion coefficient (m^2^/s)	2.5 × 10^−13^@85 °C/85% RH 7.2 × 10^−14^@60 °C/60% RH (From this study)	1.65 × 10^−12^@85 °C/85% RH 6.375 × 10^−13^@60 °C/60% RH * [21]	0	0
Saturation concentration (mol/m^3^)	220.2@85 °C/85% RH 212.9@60 °C/60% RH (From this work)	574.4@85 °C/85% RH 368.5@60 °C/60% RH * [21]	0	0

* Extrapolation from the results in this paper [21].

**Table 2:** Summary of FEM cases in this study.

	EMC Thickness	PCB Thickness	85 °C/85% RH	60 °C/60% RH	Thermal Expansion	Hygroscopic Swelling
Case 1	3.7 mm	1.7 mm	√	-	√	√
Case 2	3.7 mm	1.7 mm	-	√	√	√
Case 3	3.7 mm	1.7 mm	√	-	√	-
Case 4	3.7 mm	1.7 mm	√	-	-	√
Case 5	2.7~3.7 mm	1.7 mm	√	-	√	√
Case 6	3.7 mm	0.7~1.7 mm	√	-	√	√

**Table 3:** Moisture diffusion coefficients and saturation concentrations of the new commercial EMC at different conditions.

Test Conditions	D (m^2^/s)	C (mol/m^3^)
85 °C/85% RH	2.5 × 10^−13^	220.2
60 °C/60% RH	7.2 × 10^−14^	212.9

## Data Availability

Not applicable.
